# Species-Specific Responses of Bloom-Forming Algae to the Ocean Warming and Acidification

**DOI:** 10.3390/plants13172433

**Published:** 2024-08-30

**Authors:** Hailong Wu, Fangsheng Cheng, Jiang Chen, He Li, Juntian Xu, Peimin He, Sufang Li

**Affiliations:** 1 Jiangsu Key Laboratory of Marine Bioresources and Environment, Jiangsu Ocean University, Lianyungang 222005, China; 2022220803@jou.edu.cn (F.C.);; 2 Co-Innovation Center of Jiangsu Marine Bio-Industry Technology, Jiangsu Ocean University, Lianyungang 222005, China; 3 College of Marine Ecology and Environment, Shanghai Ocean University, Shanghai 201306, China

**Keywords:** bloom-forming macroalgae, growth, increased temperature, enriched pCO_2_, physiological responses

## Abstract

Macroalgal biomass blooms, including those causing the green and golden tides, have been rising along Chinese coasts, resulting in considerable social impacts and economic losses. To understand the links between the ongoing climate changes (ocean warming and acidification) and algal tide formation, the effects of temperature (20 and 24 °C), pCO_2_ concentration (Partial Pressure of Carbon Dioxide, 410 ppm and 1000 ppm) and their interaction on the growth of *Ulva prolifera* and *Ulva lactuca* (green tide forming species), as well as *Sargassum horneri* (golden tide forming species) were investigated. The results indicate that the concurrent rises in temperature and pCO_2_ level significantly boosted the growth and nutrient uptake rates of *U. lactuca*. For *U. prolifera*, the heightened growth and photosynthetic efficiency under higher CO_2_ conditions are likely due to the increased availability of inorganic carbon. In contrast, *S. horneri* exhibited negligible responsiveness to the individual and combined effects of the increased temperature and CO_2_ concentration. These outcomes indicate that the progressive climate changes, characterized by ocean warming and acidification, are likely to escalate the incidence of green tides caused by *Ulva* species, whereas they are not anticipated to precipitate golden tides.

## 1. Introduction

The ongoing emission of greenhouse gases due to human activities such as burning fossil fuels and deforestation has raised the atmospheric CO_2_ concentration from 280 ppm in pre-industrial times to 410 ppm at present, with the prediction that it will be more than twice the pre-industrial concentration by 2100 [1]. The increase not only enriches dissolved CO_2_ in the ocean, resulting in a pH reduction (ocean acidification, OA), but also elevates the average seawater surface temperature, accelerating ocean warming [2,3]. Alterations in the physical and chemical properties of seawater, driven by climate changes, influence the calcification rates, demography, abundance, distribution and adaptability of marine organisms [4,5]. Macroalgae serve a crucial role in maintaining ocean ecosystem biodiversity and stability since they offer food, habitat, and nursery for other marine life [6,7,8]. Exposure to fluctuating environments typically affects their growth, photosynthetic activity, biochemical composition and even reproductive pattern [9,10].

Most algae are sensitive to the changes in CO_2_ availability, as both the dissolved inorganic carbon (DIC) and pH can impact their photosynthetic performance [11]. Indeed, photosynthetic carbon (C) fixation is primarily regulated by the enzyme Ribulose-1,5-biophosphate carboxylase/oxygenase (Rubisco), which exclusively utilizes CO_2_ [12] and is pH dependent [13]. The species-specific responses of marine algae to CO_2_ have been extensively documented and are largely attributed to their diverse strategies for inorganic carbon (Ci) acquisition [14]. Regarding most algae, the current CO_2_ level saturates their photosynthesis because they may actively use bicarbonate (HCO_3_^−^) or directly uptake CO_2_ using carbon-concentration mechanisms (CCMs) [15]. Nevertheless, insufficient HCO_3_^−^ usage or CO_2_ acquisition would restrict the photosynthetic rate, consequently slowing down algal growth. 

Some seaweeds, such as *Saccharina japonica* [7,11] and *Ulva* species [16], benefit from enriched pCO_2_. The mechanism rates of these algae under the current CO_2_ levels were limited by insufficient carbon acquisition but accelerated by the enriched conditions. On the other hand, algae such as *Gracilaria lemaneiformis* [17] and *Pyropia haitanensis* [14] were negatively impacted by increased CO_2_ levels. The decreased pH is typically attributed to the adverse impacts because it may induce the ROS (reactive oxygen species) that damage photosystems and inhibit the activity of the enzymes that are involved in carbon assimilation [11,18]. Furthermore, other algae such as *Alaria esculenta* and *Sargassum horneri* have been reported to be unaffected by elevated pCO_2_, and the neutral effect could be linked to their long-term adaptation to the acidification stress [19,20]. 

Temperature is another crucial factor affecting algal growth and physiology. Several primary sites of temperature sensitivity, such as PSII (in particular the D1 protein), the thylakoid membrane and the photosynthetic apparatus (the attacking sites of ROS), as well as the enzymes involved in the Calvin–Benson cycle, have been proposed [21]. For instance, an increased temperature may induce the incomplete oxidation of water at the electron donor side of PSII, resulting in an accumulation of H_2_O_2_, which would be then reduced by manganese to a highly oxidizing HO· through the Fenton reaction [22]. Nevertheless, previous studies have observed that elevated temperatures had no influence or positively enhanced algal photosynthesis, respiration and growth at elevated temperatures, indicating that the temperatures studied are still within the sub-optimal range for these photoautotrophs [2,14,19,20].

*Ulva* species and *S. horneri* have been identified as key contributors to global green and golden tide events, respectively [23]. Their remarkable tolerance to diverse environmental conditions, coupled with the rapid growth rates, has made them global problematic agents [24,25]. These macroalgal blooms pose a multifaceted threat to marine and estuarine ecosystems, leading to ecological disturbances such as hypoxia, the emission of toxic hydrogen sulfide harmful to aquatic life and humans, and the loss of species that are crucial for ecological balance and economic value [23]. 

Elevated temperatures increase the CO_2_ diffusion coefficient and reduce its viscosity, both of which elevate the Ci availability for algae [19]. Therefore, it is imperative to examine the synergistic effects of these factors on macroalgae. Although studies have shown the separate and combined effects of CO_2_ and temperature on macroalgae [2,7,14,20], the specific impacts of these factors on bloom-forming genera like *Ulva* and *Sargassum* are not fully understood. This study, accordingly, investigated the independent and interactive impacts of CO_2_ and temperature on the growth, physiological traits and bio-composition of *U. prolifera*, *U. lactuca* and *S. horneri*, aiming to clarify the link between the outbreaks of green/golden tides and the progressive climate changes.

## 2. Results

### 2.1. Growth

**Figure 1:**
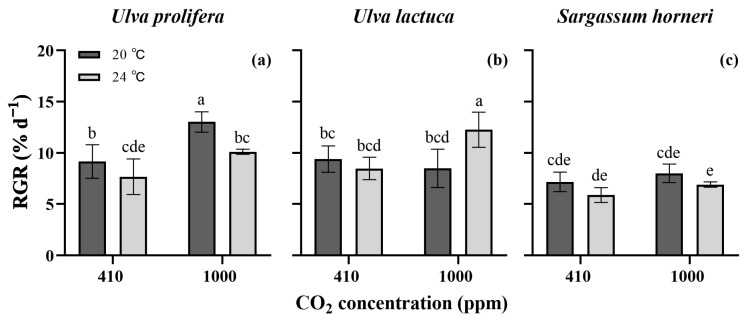
Relative growth rates (RGRs) of *Ulva prolifera* (**a**), *Ulva lactuca* (**b**) and *Sargassum horneri* (**c**) grown at different pCO_2_ levels and temperatures. All the results are shown as mean value ± SD (*n* = 3). Different letters indicate significant differences (*p* < 0.05) using Scheffe’s post-hoc test.

The growth of the three bloom-forming macroalgae, including two green algae, *U. prolifera* and *U. lactuca*, and one brown alga, *S. horneri*, was markedly affected by pCO_2_ (*p* < 0.001, three-way ANOVA, Table S1, Supplementary Materials). The temperature and its combination with pCO_2_ had no effect on the RGR (*p* > 0.05). The RGR varied among species (*p* < 0.001) and donated the maximum effect size (η^2^ = 0.638, *p* < 0.001, Table S1, Supplementary Materials) and had a significant influence with pCO_2_ and temperature (*p* < 0.01). Overall, the two green algae grew faster than *S. horneri* (Figure 1). At 20 °C, *U. prolifera* showed a higher RGR (1.4 times, Figure 1a) under the enriched CO_2_ level than that under ambient conditions. However, there was little significance at 24 °C. A remarkable increase in the RGR was found in *U. lactuca* when both the temperature and CO_2_ (greenhouse conditions, GH conditions) were simultaneously increased (Figure 1b). The growth of *S. horneri* was unaffected by the two environmental factors and their interaction (Figure 1c).

### 2.2. Pigment Contents

**Figure 2:**
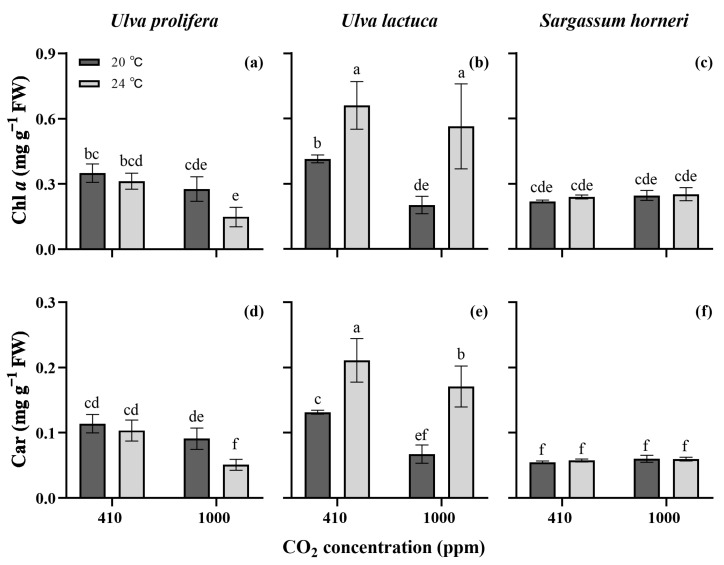
Pigment contents of *Ulva prolifera* (**a**,**d**), *Ulva lactuca* (**b**,**e**) and *Sargassum horneri* (**c**,**f**) grown at different pCO_2_ levels and temperatures. All the results are shown as mean value ± SD (*n* = 3). Different letters indicate significant differences (*p* < 0.05) using Scheffe’s post-hoc test.

Both the Chl *a* and Car contents varied among the three algae (*p* < 0.001) and were significantly affected by the pCO_2_, temperature and the interactions between species and the other two factors (*p* < 0.05, three-way ANOVA, Table S1, Supplementary Materials), whereas the interplay between pCO_2_ and temperature, as well as their combination with species, had no impact on pigment contents (*p* > 0.05). The algal species exhibited the maximum effects (η^2^ = 0.734 and 0.881, respectively) on Chl *a* and Car contents. *S. horneri* had low pigment contents (around 0.24 mg Chl *a* g^−1^ FW and 0.06 mg Car g^−1^ FW) and no significance was observed among all environmental treatments (*p* > 0.05, Figure 2). The higher temperature decreased the pigment contents of *U. prolifera* under the enriched CO_2_ conditions, in particular under the GH condition. Contrarily, the higher temperature obviously raised the pigment contents of *U. lactuca* under the two CO_2_ concentrations, but the Chl *a* and Car contents showed decreases when the pCO_2_ level increased. The highest pigment amounts were observed in *U. lactuca* grown at 24 °C under the ambient pCO_2_ conditions, which were 0.66 mg Chl *a* g^−1^ FW and 0.21 mg Car g^−1^ FW.

### 2.3. Photosynthetic Rate

**Figure 3:**
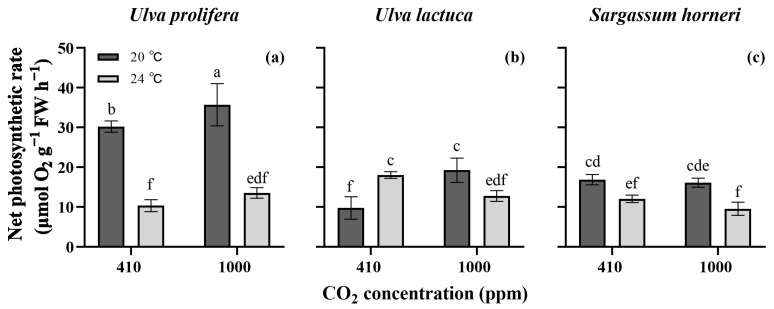
Net photosynthetic rates of *Ulva prolifera* (**a**), *Ulva lactuca* (**b**) and *Sargassum horneri* (**c**) grown at different pCO_2_ levels and temperatures. All the results are shown as mean value ± SD (*n* = 3). Different letters indicate significant differences (*p* < 0.05) using Scheffe’s post-hoc test.

Consistent with the other parameters, the species had a high significant effect on the photosynthetic rate (η^2^ = 0.817, *p* < 0.001, three-way ANOVA, Table S1, Supplementary Materials). The CO_2_, temperature, their interactions and the interaction with species impaired the *P_n_* significantly (*p* < 0.05). *U. prolifera* showed a higher net photosynthetic rate at 20 °C than at elevated temperatures (*p* < 0.05, Figure 3), and the maximal *P_n_* was under the higher CO_2_ level conditions and was 35.7 μmol O_2_ g^−1^ FW h^−1^. The enriched pCO_2_ increased the *P_n_* of *U. lactuca* at 20 °C but reduced it at 24 °C (*p* < 0.05). Under atmospheric CO_2_ levels, the elevated temperature increased the *P_n_*, whereas under the enriched CO_2_ conditions it decreased the *P_n_*. The *P_n_* of *S. horneri* displayed no variations between the two CO_2_ levels (*p* > 0.05) but decreased when the temperature was elevated (*p* < 0.05). 

### 2.4. Nutrients Uptake Rates

**Figure 4:**
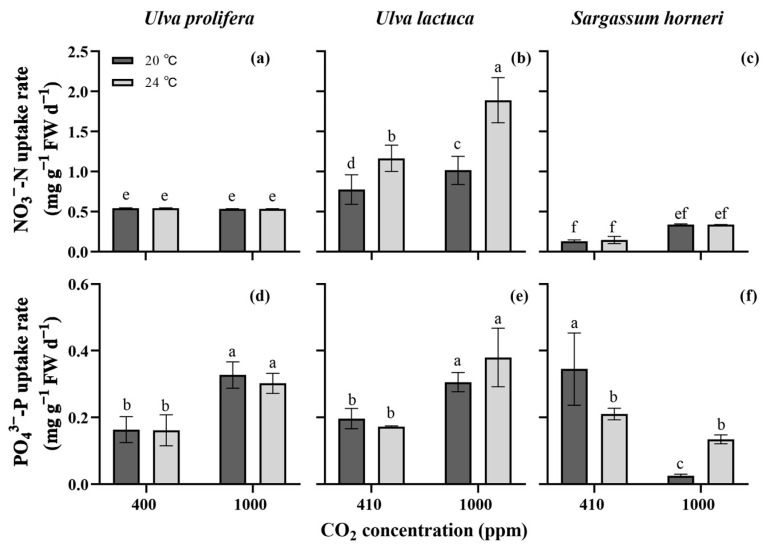
Nitrogen (**a**–**c**) and phosphorus uptake rates (**d**–**f**) of *Ulva prolifera*, *Ulva lactuca* and *Sargassum horneri* grown under different pCO_2_ levels and temperatures. All the results are shown as mean value ± SD (*n* = 3). Different letters indicate significant differences (*p* < 0.05) using Scheffe’s post-hoc test.

The three seaweeds displayed completely different N removal behaviors (η^2^ = 0.945, *p* < 0.001, three-way ANOVA, Table S1, Supplementary Materials), which were also significantly impacted by CO_2_, temperature and their combinations with species (*p* < 0.05). Figure 4a–c show that the green alga *U. lactuca* absorbed nitrogen faster than *U. prolifera* and *S. horneri* (*p* < 0.05). The brown alga had the lowest N uptake rates (0.13–0.14 mg g^−1^ FW d^−1^). Specifically, there was no significant effect of CO_2_ and temperature on the N uptake rates of both *U. prolifera* and *S. horneri*. Elevated pCO_2_ values and temperatures accelerated the N uptake of *U. lactuca*, and the maximal rate (1.9 mg g^−1^ FW d^−1^) was observed under the GH conditions.

Species and CO_2_ level showed individual and synergistic effects on the P uptake of the three seaweeds (*p* < 0.05, three-way ANOVA, Table S1, Supplementary Materials), but the temperature and its combination with species had no significance (*p* > 0.05). In addition, pCO_2_ combined with temperature, and their combination together with species revealed notable cooperative influences on P uptake (*p* < 0.01). Elevated pCO_2_ levels increased the P uptake rates of green algae regardless of temperature but reduced the P uptake rate of *S. horneri* (*p* < 0.05, Figure 4d–f). Concurrently, higher temperatures were observed to decrease the P uptake rate of *S. horneri* under ambient CO_2_ conditions, whereas increasing it under the enriched CO_2_ conditions (*p* < 0.05, Figure 4f).

### 2.5. Soluble Protein Content

**Figure 5:**
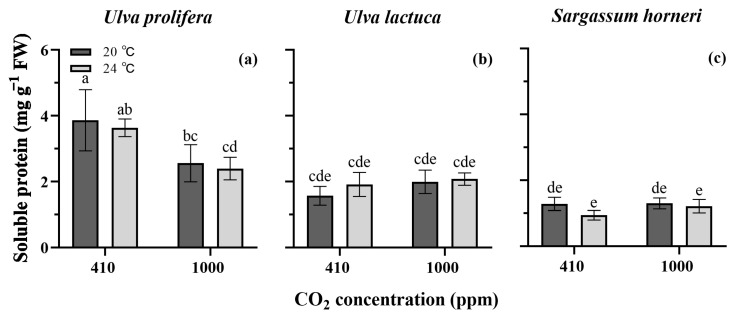
Soluble protein contents of *Ulva prolifera* (**a**), *Ulva lactuca* (**b**) and *Sargassum horneri* (**c**) grown at different pCO_2_ levels and temperatures. All the results are shown as mean value ± SD (*n* = 3). Different letters indicate significant differences (*p* < 0.05) using Scheffe’s post-hoc test.

Figure 5 shows the soluble protein (SP) contents of *U. prolifera*, *U. lactuca* and *S. horneri*. The species and CO_2_ individually and interactively affected the SP content (*p* < 0.05), whereas the temperature and its interactions with species and pCO_2_ had little effect on the SP content (*p* > 0.05, three-way ANOVA, Table S1, Supplementary Materials). *U. prolifera* showed the highest SP content, followed by *U. lactuca* and then *S. horneri*. CO_2_ levels and temperature exhibited no effect on the latter two algae, but the elevated pCO_2_ inhibited the SP accumulation of *U. prolifera* (*p* < 0.05).

## 3. Discussion

The growth of the three algae species was differentially influenced (Figure 1), indicating the different responses of the bloom-forming algae to the ongoing climate changes. The diversity in responses may be due to their distinct acclimation strategies, such as the regulation of energy and carbon partitioning via photosynthesis and respiration. These strategies enable organisms to optimize their growth and survival under varying environmental conditions [19,26]. In our study, the green algae grew faster than the Phaeophyceae, suggesting a higher potential for green tide outbreaks caused by *Ulva* species compared to the golden tides caused by *S. horneri*. This could be linked to the higher content of antenna pigments in green algae (except the *U. prolifera* grown under the GH conditions, Figure 2), which may absorb more photons for biomass gains [27]. In addition, the higher nutrient uptake rates may accelerate the growth of *Ulva* species, as they are crucial for the synthesis of essential biomolecules such as proteins, enzymes, energy-transfer molecules (ATP, ADP), chlorophylls and genetic materials (RNA, DNA) [28]. Shahar et al. [29] also confirmed that the higher daily growth rate of *Ulva fasciata* was associated with its higher N utilization efficiency.

Under enriched CO_2_ conditions, *U. prolifera* showed increased RGRs at both temperatures, mainly due to the increased DIC availability. Appropriately elevated CO_2_ levels have been reported to enhance macroalgal photosynthetic activities, providing the energy and carbon needed for biomolecule synthesis and biomass accumulation [7,14,16]. This study confirmed this finding, with an increased net photosynthetic rate (*P_n_*) observed in *U. prolifera* under the enriched pCO_2_ conditions (Figure 3a). In addition to the increased *P_n_*, the enhanced P uptake rate may also contribute to the greater growth potential (Figure 4d), given that phosphorus facilitates the storage and exchange of energy and information in cells [30]. However, it is noteworthy that enriched pCO_2_ declined the contents of photosynthetic pigments, despite only minor reductions in Chl *a* and Car being observed at 20 °C (Figure 2). A high pCO_2_ level generally raises the HCO_3_^−^ concentration, driving the CCMs with a lowered energy demand, which may result in a decrease in the biosynthesis of energy-capturing pigments. This phenomenon of “pigment economy” has been reported in some *Ulva* species by Gao et al. [31] and Wang et al. [16]. Consistent with those studies and same results of *U. prolifera* in this work, reductions in the pigment contents induced by the elevated pCO_2_ were also observed in *U. lactuca* (Figure 2b,e).

At the higher temperature, *U. prolifera* exhibited reduced RGRs, although the GH conditions partly offset this decline. The lowered photosynthetic rate at 24 °C is likely a primary reason for the RGR reduction. Cui et al. [32] identified 20 °C as the optimal temperature for *U. prolifera*, noting that the growth rate decreased significantly at 25 °C. High temperatures can lead to the accumulation of ROS and oxidative stress, which may damage the photosynthetic apparatus. Muñoz et al. [33] suggested that the detrimental effects of elevated temperatures on *Lithothamnion crispatum* and *Sonderophycus capensis* may stem from a significant rise in ROS levels. Therefore, the decreased growth rate and *P_n_* observed in this study could be attributed to the increased temperature exceeding the algal thermal tolerance threshold. 

On the other hand, temperature had little influence on the soluble protein (SP) content of *U. prolifera*, whereas under the elevated CO_2_ and GH conditions, the SP contents were significantly reduced (Figure 5a). This suggests that the CO_2_ level exerted a more pronounced effect on SP accumulation. Suárez-Álvarez et al. [34] proposed that the reduction in soluble protein content at a high CO_2_ level may be due to an uncoupling of carbon assimilation via photosynthesis with an increased nitrogen demand. In this work, although elevated pCO_2_ increased the *P_n_*, there was little effect on nitrogen uptake (Figure 4a), potentially leading to changes in nitrogen distribution among proteins and other nitrogen-containing compounds [34,35]. Additionally, the reduction in the soluble protein content under the enriched CO_2_ conditions might also be associated with a reallocation of energy towards faster growth.

In the case of *U. lactuca*, an increased temperature alone showed no influence on algal growth. However, the RGR under GH conditions was notably higher, approximately 1.4 times greater than that under both the current conditions and the combination of enriched CO_2_ with 20 °C (Figure 1b). This suggests that the predicted climate changes may potentially increase the outbreak frequency of green tides caused by *U. lactuca*. Under GH conditions, the increased temperature increases the diffusive coefficient of CO_2_, thereby facilitating greater CO_2_ availability for biomass accumulation [19,36]. Similar increases in algal biomass under GH conditions have been documented in *Gracilariopsis lemaneiformis* [37], *Pycodry rubens* and *Saccorhiza dermatodea* [19]. 

*U. lactuca* exhibited a higher pigment content at 24 °C than at 20 °C under both pCO_2_ conditions (Figure 2). The increased pigment accumulation might serve to mitigate the oxidative stress in the photosynthetic apparatus induced by the higher temperature [14,38]. The rises in the Chl *a* and Car contents were also closely correlated with the higher N uptake rate under enriched CO_2_ conditions (Figure 4). This may be because cellular nitrogen plays important roles in the accumulation of photosynthetic pigments. Similar findings have been reported in *Kappaphycus alvarezii* by Peter et al. [25]. However, compared to at 24 °C under ambient CO_2_ conditions, the elevated pigment contents under GH conditions appears to contradict the observed decline in the photosynthetic rate (Figure 2 and Figure 3). We hypothesize that the further decreased intracellular pH under GH conditions might adversely interrupt the electron donor side of PSII centers, reducing the oxygen evolution rate (Figure 3b), which is in line with the finding observed by Schlodder and Meyer [39]. However, to clearly elucidate the lack of correlation between pigment contents and photosynthetic performance, further studies should be carried out. 

*S. horneri* demonstrated lower growth rates than the two *Ulva* species, and the elevated temperature and enriched pCO_2_, both independently and interactively, had little impact on the growth, pigment contents, N uptake and soluble protein content, though the increased temperature slightly decreased the *P_n_*. This may suggest that compared to the *Ulva* species, *S. horneri* is less sensitive to climate changes. The *Ulva* genus tends to bloom in the photic zone of eutrophic coasts and estuaries, whereas the pelagic *Sargassum* genus is widespread in the ocean. In recent years, however, unusual bimacroalgal blooms have been observed in the Yellow Sea of China [23]. Given the proximity of the sampling sites for the three algae, the possibility of green and golden tides occurring simultaneously in the maritime area of Jiangsu Province could not be ruled out (See Section 4.1). Yet, the findings revealed the green algae showed incomparable performance in growth and physiological characteristics compared to the brown alga; therefore, it appears more likely that the outbreak frequency of green tides at the studied location will be higher.

In our previous study [20], however, the increased temperature alone and its combination with the elevated pCO_2_ enhanced the growth, photosynthesis and carbon assimilation of *S. horneri*. The different responses in the two assays may be linked to the sampling of *S. horneri* at different growth stages. In the previous assay, *S. horneri* was collected in March, during its rapid growth phase, whereas in the current study, the sampling was performed in June, corresponding to the maturation phase of this alga [40]. Mature *S. horneri* exhibits greater resilience to the fluctuating climate changes, suggesting that the greenhouse effect may exacerbate golden tides during the algal growth stage but may have negligible influence during its maturation stage.

On the whole, the experimental results provide evidence that the three bloom-forming algae investigated here displayed distinct responses to the ongoing climate changes in terms of the increasing atmospheric CO_2_ and temperature. The enriched pCO_2_ and elevated temperature synergistically raised the growth rate, pigment content and nutrient uptake rates of *U. lactuca*, and higher pCO_2_ concentrations increased the growth and photosynthetic rates of *U. prolifera*. However, those factors showed little significance in *S. horneri*. We propose that the combined increase in temperature and CO_2_ concentration would aggravate the outbreaks of green tides formed by *U. lactuca*, while CO_2_ enrichment may be specifically associated with the blooms of *U. prolifera*, and that the factors tested may have no link to the biomass blooms of *S. horneri*.

## 4. Materials and Methods

### 4.1. Sample Collection

*U. prolifera* and *U. lactuca* were collected in June 2021 from Gaogong Island (34°54′31″ N; 119°31′57″ E), Lianyungang, Jiangsu Province, China; *S. horneri* was obtained from Qidong, Nantong (31°41′6″ N; 121°25′40″ E), Jiangsu Province, China. The temperatures at the sampling sites were 18–20 °C. The samples were placed in a closed tank containing ice packs and transferred to the laboratory within 2 h. Healthy thalli were selected and rinsed with sterilized seawater and, in particular, the secondary branches of *S. horneri* were cut into around 2.0 cm. The thalli were stock-cultured in three Erlenmeyer flasks (5 L) containing 5 L autoclaved seawater in the incubators (GXZ-500C, Ningbo Jiangnan instrument factory, China) and maintained at 20 °C with air-filtered aeration and an irradiance of 100 μmol photon m^−2^ s^−1^ (12:12 h light/dark cycle) for 3 days to reduce the negative effects of cutting. 

### 4.2. Experimental Design

Under the global warming process, it is projected that temperature will rise by 4 °C and the atmospheric CO_2_ concentration will reach 1000 ppm by the end of 21st century [41]. Therefore, two temperatures (20 °C and 24 °C) and two CO_2_ concentrations (410 ppm and 1000 ppm) were maintained by the incubators to study the effects of temperature, CO_2_ and their interaction on the growth and physiological traits of the three bloom-forming macroalgae. Herein, four treatments, including 20 °C + 410 ppm pCO_2_, 20 °C + 1000 ppm pCO_2_, 24 °C + 410 ppm pCO_2_ and 24 °C + 1000 ppm pCO_2_, were investigated. Each treatment was performed in three replicates. Uniformly growing and healthy 0.5 g thalli were introduced into round bottles that contained 500 mL autoclaved seawater (AS, around 30 μM N, ambient level; enriched with 8 μM P to avoid P restriction) at a starting biomass density of 1 g L^−1^. The seaweeds were continuously illuminated with 100 μmol photon m^−2^ s^−1^ (12:12 h light/dark cycle), and the medium was aerated and replaced with fresh AS every three days. In total, the seaweeds were cultured for 12 days. The fresh thalli were harvested when renewing the medium to characterize the growth rate, pigment content, nutrient uptake rates, photosynthesis and respiration rates and soluble protein content.

### 4.3. Growth Rate

The relative growth rate (RGR) was calculated using the formula RGR (% d^−1^) = 100 × Ln (W_t_/W_0_)/t, where W_t_ and W_0_ are the fresh weight (FW, g) measured at day t and the beginning of experiment, respectively [42].

### 4.4. Biochemical Components

Chlorophyll *a* (Chl *a*) and Carotenoids (Cars) were extracted using approximately 0.02 g fresh thalli. The tissue was fully submerged in 5 mL methanol and then incubated at 4 °C for 24 h in darkness. The extracts were spectrophotometrically detected at 470, 652 and 665 nm using an ultraviolet absorption spectrophotometer (U-2900, HITACHI, Tokyo, Japan). The pigment contents (mg g^−1^ FW) were estimated using the formula reported by Wellburn [43]. 

The soluble protein (SP) content (mg g^−1^ FW) was quantified using Coomassie Brilliant Blue G-250 dye according to Kochert [44]. At the end of the cultivation, 0.02 g tissue was ground in a cold mortar with phosphoric acid buffer (PBS, stored at 4 °C). The homogenate was diluted to 10 mL with PBS and centrifuged at 5000 rpm for 15 min (4 °C). A total of 1 mL supernatant was mixed with 4 mL G-250 dye solution and, after 5 min, the absorbance at 595 nm was recorded using the spectrophotometer. Bovine serum albumin was adapted as the standard (y = 0.0014 x − 0.0036, R^2^ = 0.9928).

### 4.5. Photosynthetic Oxygen Evolution

A Clark-type oxygen electrode (YSI Model 5300; Yellow Springs Instrument Co., Yellow Springs, OH, USA) was adapted to quantify the photosynthetic oxygen evolution. The thalli were cut into 1 cm length pieces and re-cultured for 1 h to reduce the mechanical damage. A total of 0.02 g of small thalli were placed into a column filled with 8 mL medium. The net photosynthetic rate (*P_n_*) was determined under the growth conditions (combined temperatures and pCO_2_ levels).

### 4.6. Nutrient Uptake Rates

At the time of the last water change, 5 mL fresh and 5 mL algae-cultured medium were collected at day 9 and at day 12, respectively. The N and P concentrations (mg L^−1^) in water samples were quantified using a nutrient analyzer (SEAL QuAAtro 39-SFA, Mequon, WI, USA), and the nutrient uptake rates were estimated using the formula [45]:

N_uptake_ (mg g^−1^ FW d^−1^) = (N_0_ − N_t_) × V/M/t (1)

where N_uptake_ represents the N (or P) uptake rate of the algae; N_0_ and N_t_ are the N (or P) concentrations in the fresh and algae-cultured media, respectively; V is the culture volume (here V = 0.5 L); M stands for biomass in the bottles; and t is the water change interval (here t = 3 d).

### 4.7. Data Analysis

All treatments were conducted in three independent biological replicates, and the data are reported as mean values with standard deviations (Mean ± SD). The relative growth rates, pigment contents, photosynthetic rates, nutrients uptake rates and soluble protein contents between the two temperatures, two pCO_2_ levels and among the three species were statistically analyzed by three-way ANOVA using IBM SPSS Statistics 26.0 software (SPSS Inc., Chicago, IL, USA). Scheffe’s multiple comparisons procedure was performed after the tests of normality and variance homogeneity. Significant variations at a level of *p* < 0.05 are shown with different letters.

## Data Availability

The data from this study are available from the corresponding author upon reasonable request.

## Supplementary Materials

The following supporting information can be downloaded at: https://www.mdpi.com/article/10.3390/plants13172433/s1, Table S1: Analysis of three-way ANOVA showing the effects of species, pCO_2_, temperature and their interactions on the relative growth rate (RGR), pigments contents, net photosynthetic rate, nitrogen and phosphorous removal rate, as well as soluble protein content of three macroalgae, *Ulva prolifera*, *Ulva lactuca* and *Sargassum horneri*.
