# Supplementary material for: Species-Specific Responses of Bloom-Forming Algae to the Ocean Warming and Acidification
Source: Plants (Basel). 2024 Aug 30;13(17):2433. doi: 10.3390/plants13172433 (PMC11396949; doi:10.3390/plants13172433)
Supplement: Supplementary file 1 [file plants-13-02433-s001.zip › plants-3128470-supplementary.pdf]

## Supplementary Materials for the article:

# Species-Specific Responses of Bloom-Forming Algae to the Ocean Warming and Acidification

**Hailong Wu [1,2,*], Fangsheng Cheng [1,2], Jiang Chen [1,2], He Li [1,2], Juntian Xu [1,2], Peimin He [2,3] and Sufang Li [1,2,*]**

[1] Jiangsu Key Laboratory of Marine Bioresources and Environment, Jiangsu Ocean University, Lianyungang 222005, China; 2022220803@jou.edu.cn (F.C.)
[2] Co-Innovation Center of Jiangsu Marine Bio-industry Technology, Jiangsu Ocean University, Lianyungang 222005, China
[3] College of Marine Ecology and Environment, Shanghai Ocean University, Shanghai 201306, China
* Correspondence: hlwu@jou.edu.cn (H.W.); lisuf@jou.edu.cn (S.L.)

**Table S1.** Analysis of of Three-way ANOVA showing the effects of species, $pCO_2$, temperature and their interactions on the relative growth rate (RGR), pigments contents, net photosynthetic rate, nitrogen and phosphorous removal rate, as well as soluble protein content of three macroalgae, *Ulva prolifera*, *Ulva lactuca* and *Sargassum horneri*.

| **parameter** | **df** | **F** | ***p*** | **Partial $\eta^2$** |
|---|---|---|---|---|
| **Relative growth rate** | | | | |
| Species | 2 | 31.758 | < **0.001** | **0.638** |
| $pCO_2$ | 1 | 18.652 | < **0.001** | 0.341 |
| Temperature | 1 | 2.369 | 0.133 | 0.062 |
| Species *$pCO_2$ | 2 | 9.093 | **0.001** | 0.336 |
| Species * Temperature | 2 | 14.817 | < **0.001** | 0.451 |
| $pCO_2$ * Temperature | 1 | 1.566 | 0.219 | 0.042 |
| Species * $pCO_2$ * Temperature | 2 | 6.514 | **0.004** | 0.266 |
| | | | | |
| **Chlorophyll *a*** | | | | |
| Species | 2 | 33.081 | < **0.001** | **0.734** |
| $pCO_2$ | 1 | 12.394 | **0.002** | 0.341 |
| Temperature | 1 | 10.547 | **0.003** | 0.305 |
| Species *$pCO_2$ | 2 | 4.918 | **0.016** | 0.291 |
| Species * Temperature | 2 | 23.532 | < **0.001** | 0.662 |
| $pCO_2$ * Temperature | 1 | 4.00E-03 | 0.948 | 0.000 |
| Species * $pCO_2$ * Temperature | 2 | 1.581 | 0.227 | 0.116 |
| | | | | |
| **Carotenoids** | | | | |
| Species | 2 | 89.005 | < **0.001** | **0.881** |
| $pCO_2$ | 1 | 28.338 | < **0.001** | 0.541 |
| Temperature | 1 | 17.408 | < **0.001** | 0.420 |
| Species *$pCO_2$ | 2 | 9.634 | **0.001** | 0.445 |
| Species * Temperature | 2 | 42.969 | < **0.001** | 0.782 |
| $pCO_2$ * Temperature | 1 | 0.069 | 0.795 | 0.003 |
| Species * $pCO_2$ * Temperature | 2 | 2.064 | 0.149 | 0.147 |
| | | | | |
| **Net photosynthetic rate** | | | | |
| Species | 2 | 53.738 | < **0.001** | 0.817 |
| $pCO_2$ | 1 | 4.589 | **0.043** | 0.161 |
| Temperature | 1 | 132.283 | < **0.001** | 0.846 |
| Species *$pCO_2$ | 2 | 5.399 | **0.012** | 0.310 |
| Species * Temperature | 2 | 75.512 | < **0.001** | **0.863** |

| | | | | |
|---|---|---|---|---|
| $pCO_2$ * Temperature | 1 | 17.394 | < **0.001** | 0.420 |
| Species * $pCO_2$ * Temperature | 2 | 8.026 | **0.002** | 0.401 |
| | | | | |
| **N removal rate** | | | | |
| Species | 2 | 206.564 | < **0.001** | **0.945** |
| $pCO_2$ | 1 | 31.232 | < **0.001** | 0.565 |
| Temperature | 1 | 28.457 | < **0.001** | 0.542 |
| Species *$pCO_2$ | 2 | 12.724 | < **0.001** | 0.515 |
| Species * Temperature | 2 | 27.471 | < **0.001** | 0.696 |
| $pCO_2$ * Temperature | 1 | 3.789 | 0.063 | 0.136 |
| Species * $pCO_2$ * Temperature | 2 | 4.214 | **0.027** | 0.260 |
| | | | | |
| **P removal rate** | | | | |
| Species | 2 | 9.84 | **0.001** | 0.451 |
| $pCO_2$ | 1 | 5.429 | **0.029** | 0.184 |
| Temperature | 1 | 0.001 | 0.980 | 0.000 |
| Species *$pCO_2$ | 2 | 53.745 | < **0.001** | **0.817** |
| Species * Temperature | 2 | 0.619 | 0.547 | 0.049 |
| $pCO_2$ * Temperature | 1 | 10.985 | **0.003** | 0.314 |
| Species * $pCO_2$ * Temperature | 2 | 5.759 | **0.009** | 0.324 |
| | | | | |
| **Soluble protein content** | | | | |
| Species | 2 | 73.024 | < **0.001** | **0.859** |
| $pCO_2$ | 1 | 4.407 | **0.047** | 0.155 |
| Temperature | 1 | 0.245 | 0.625 | 0.010 |
| Species *$pCO_2$ | 2 | 14.311 | < **0.001** | 0.544 |
| Species * Temperature | 2 | 1.115 | 0.344 | 0.085 |
| $pCO_2$ * Temperature | 1 | 0.006 | 0.937 | 0.000 |
| Species * $pCO_2$ * Temperature | 2 | 0.326 | 0.725 | 0.026 |
